# Determining Trap Compliances, Microsphere Size Variations, and Response Linearities in Single DNA Molecule Elasticity Measurements with Optical Tweezers

**DOI:** 10.3389/fmolb.2021.605102

**Published:** 2021-03-22

**Authors:** Youbin Mo, Mounir Fizari, Kristina Koharchik, Douglas E. Smith

**Affiliations:** Department of Physics, University of California San Diego, La Jolla, CA, United States

**Keywords:** optical trap, laser tweezers, single-molecule, DNA elasticity, calibration, microsphere size, force, trap stiffness

## Abstract

We previously introduced the use of DNA molecules for calibration of biophysical force and displacement measurements with optical tweezers. Force and length scale factors can be determined from measurements of DNA stretching. Trap compliance can be determined by fitting the data to a nonlinear DNA elasticity model, however, noise/drift/offsets in the measurement can affect the reliability of this determination. Here we demonstrate a more robust method that uses a linear approximation for DNA elasticity applied to high force range (25–45 pN) data. We show that this method can be used to assess how small variations in microsphere sizes affect DNA length measurements and demonstrate methods for correcting for these errors. We further show that these measurements can be used to check assumed linearities of system responses. Finally, we demonstrate methods combining microsphere imaging and DNA stretching to check the compliance and positioning of individual traps.

## Introduction

Optical tweezers have many applications in biophysics (Moffitt et al., 2008; Fazal and Block, 2011; Jones et al., 2015; Hashemi Shabestari et al., 2017; Polimeno et al., 2018; Robertson-Anderson, 2018; Choudhary et al., 2019; Berns, 2020), with one powerful approach being single biomolecule manipulation (Wang et al., 1997; Bustamante et al., 2000a; Gemmen et al., 2006; Tsay et al., 2010; Chemla and Smith, 2012; Keller et al., 2014; Migliori et al., 2014; Pongor et al., 2017; Bustamante et al., 2020b; Lehmann et al., 2020). Here we describe several useful methods for system calibration/characterization based on single DNA molecule manipulation.

In our instrument two laser beams create two traps and the position of one is adjusted (Abbondanzieri et al., 2005; Moffitt et al., 2006; delToro and Smith, 2014; delToro, 2015). The force acting on a trapped microsphere is determined by measuring laser deflection (Smith et al., 2003). Single DNA molecules are tethered between two microspheres and we move them apart to stretch the DNA while measuring applied force (delToro and Smith, 2014). In many biophysical studies one wants to measure force and changes in the molecular length or extension due to translocation by a molecular motor or interactions with other biomolecules/ligands that induce conformational changes. In our system, several parameters need to be determined: force and trap displacement scale factors, separation offset between the traps, and trap compliances (Rickgauer et al., 2006). Conventional methods for calibrating displacement include microsphere tracking *via* calibrated imaging systems and displacement with calibrated positioning stages; and methods for calibrating force and compliance include analyses of Brownian fluctuations, applied fluid drag forces, and trapping beam momentum changes (Smith et al., 2003; Berg-Sørensen and Flyvbjerg, 2004; Neuman and Block, 2004; Tolić-Nørrelykke et al., 2006; Viana et al., 2006; Jahnel et al., 2011; Sarshar et al., 2014; Jones et al., 2015; Bui et al., 2018; Yale et al., 2018; Melo et al., 2020). We introduced an alternative approach using DNA molecules for calibration based on their known lengths and elastic properties (Rickgauer et al., 2006; delToro and Smith, 2014). This is not intended to be a more accurate method than others, but rather a complementary one that has several useful attributes: 1) all four needed calibration factors can be determined simultaneously via a single type of measurement; 2) it is relatively easy to implement, especially if one is already working with DNA; 3) it does not require a calibrated imaging system, characterization of the optical system, precise control of sample stage position, or application of fluid drag forces; 4) it is an independent calibration method that can be used to check other methods; and 5) calibration extends to high forces (45 pN) and the linearity of system responses can be assessed. Advantages of DNA are that its elasticity is well characterized (Wang et al., 1997; Bustamante et al., 2000b; Wenner et al., 2002), its length can be precisely controlled in increments of 1 basepair (0.34 nm), and particular DNAs can be exactly replicated in any lab.

We determine force scale factor based on the DNA overstretch transition that occurs at ∼64 pN in the conditions used (Smith et al., 1996; Wenner et al., 2002; delToro and Smith, 2014). Displacement scale factor is determined by measuring two DNA molecules having different lengths (Rickgauer et al., 2006; delToro and Smith, 2014). Series compliance of the traps and relative trap positions can be determined by fitting of a nonlinear DNA elastic force model to measurements, however this may not be reliable due to measurement noise and offsets, particularly in the low-force regime (Rickgauer et al., 2006; delToro and Smith, 2014). We describe here a more reliable method using high-force range data (25–45 pN) where a linearized DNA elasticity model is accurate. This method can be used to check assumed linearities of system responses and errors in DNA length measurements caused by variations in the microsphere sizes. We describe methods to correct these errors. We also describe how combined microsphere imaging can be used to check positioning and compliances of individual traps.

The concept of using DNA measurements for calibration could also be applied, with minor modifications, to other types of optical tweezers setups, magnetic tweezers, and AFM/microneedle instruments that use force-cantilevers (Neuman and Nagy, 2008), in any case where single DNA stretching can be measured. In single optical trap and cantilever systems, DNA can be attached at one end to the sample chamber surface and stretched via a piezo-actuated stage (Neuman and Nagy, 2008).

## Methods, Results, and Discussion

### Linear Approximation for DNA Elasticity

The elasticity of DNA is well described by the extensible worm-like chain (WLC) model that predicts:

$$\frac{x}{L}=\;1-\sqrt{\frac{kT}{4FP}}+\frac{F}{S}$$ (1)

where *F* is the stretching force, *x* is the DNA end-to-end extension, *L* is the unstretched DNA contour length (0.34 nm per basepair), *k* the Boltzmann constant, *T* the absolute temperature (kT≅4.14 pN nm at room temperature), *S* the DNA stretch modulus, and *P* the DNA persistence length (Marko and Siggia, 1995; Odijk, 1995; Bustamante et al., 2000b). In the conditions we use, 10 mM Tris-HCl, pH 7.5, 150 mM NaCl, *S* = 1275 pN and *P* = 45 nm according to published studies by Wenner *et al.* that consider ionic dependence (Wang et al., 1997; Wenner et al., 2002; Lipfert et al., 2014).

**FIGURE 1:**
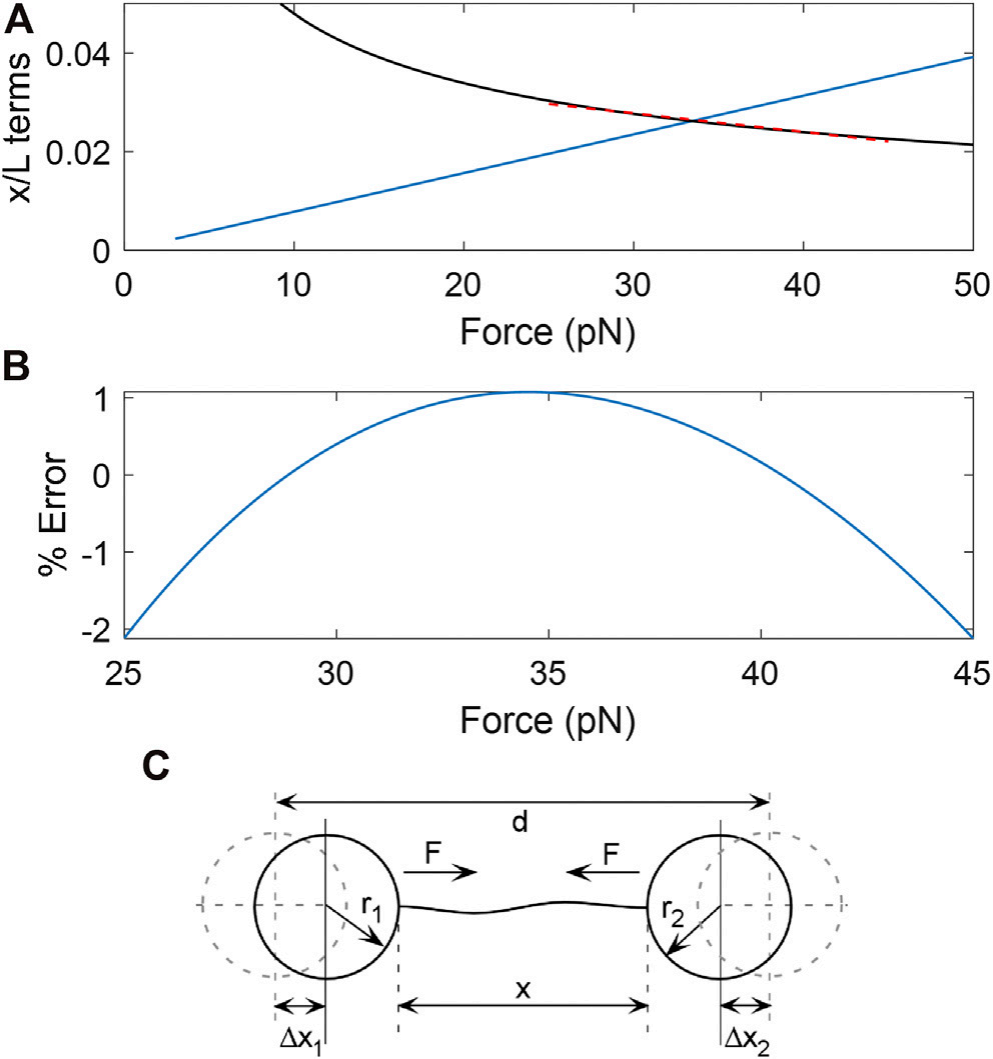
**(A)** Plots of the magnitudes of the two force-dependent terms in Eq. (1); the linear term in blue. The square-root term (black) can be accurately approximated by a line over the range from F = 25–45 pN (red dashed line). **(B)** % error made in the square-root term by the linear approximation. **(C)** Schematic illustration of the variables involved in force-extension measurements of DNA stretched between two optically trapped microspheres. The distance between the trap centers is *d*, the end-to-end extension of the DNA is *x*, the radii of the microspheres are *r*_*1*_ and *r*_*2*_, the force exerted by the tensioned DNA on the microspheres is *F*, and the displacements of the microspheres from the trap centers are *Δx*_*1*_ and *Δx*_*2*_.

A difficulty is that parameter determination via nonlinear fitting of data to this model is sensitive to experimental noise/drift/offsets, particularly at low force (delToro and Smith, 2014). However, a linear approximation of the square-root term in Eq. (1) is valid in a restricted high-force range. We use a maximum of 45 pN to stay well below the onset of non-linear behavior caused by the DNA overstretch transition (Wenner et al., 2002) and reduce the probability DNA detachment (Fuller et al., 2006). From 25 to 45 pN the square-root term can be approximated with the function *y*=(-3.78E-4)*F* + 0.0391 (Figure 1A; *F* in pN). The average error is ∼0.3% and maximum error ∼2% (Figure 1B). This results in a linearized Eq. (1):

$$\frac{x}{L}=\;A+BF$$ (2)

Where *A* = 0.961 and *B* = 1.16E-3 for the conditions we use.

### Method to Determine Trap Compliances

A single DNA molecule is stretched by increasing the separation, *d*, between the two traps as illustrated in Figure 1C. This is done by steering one of the beams using a mirror tilted by a piezoelectric actuator. Further details and a schematic diagram of the system are given in the Supplementary Materials and Ref. (delToro, 2015). The voltage applied to control the mirror actuator is referred to as *V*_*mirror*_ and the value of *V*_*mirror*_ when the two traps overlap is referred to as *V*_*overlap*_. The system is intended to have a linear response so that *d* = *β*(*V*_*mirror*_−*V*_*overlap*_). Once the calibration parameters *β* and *V*_*overlap*_ are known, the separation of the two traps in nanometers can be determined. We showed previously that the displacement scale factor *β* can be accurately determined by measuring two DNA molecules of known lengths (see Supplementary Materials and Ref. (delToro and Smith, 2014). For our system *β* = 980 nm/V. Below we will describe methods for determining *V*_*overlap*_ and checking the assumed linearity of this relationship.

Force *F = αV*_*PSD*_ is determined by measuring laser deflections with a position-sensing detector (PSD), where *V*_*PSD*_ is the detector signal and *α* the force scale factor. We showed previously that *α* can be accurately determined by measurements of the DNA overstretch transition (see Supplementary Materials and Refs. (delToro and Smith, 2014; Farré et al., 2010). For our system *α* = 38.3 pN/V. Below we will discuss a method for checking the linearity of this relationship. Note that in a dual-trap system *F* can be measured with either trap. DNA is stretched under tension between the two microspheres, so the magnitude of the force acting on each is the same.

In the Hookean regime a trapped microsphere subject to force *F* is displaced from its equilibrium position by *Δx = γ′F*, where γ′ is the trap compliance (Polimeno et al., 2018). The two traps may have different compliances *γ*_*1*_ and *γ*_*2*_, but determination of the series compliance *γ = γ*_*1*_ *+ γ*_*2*_ is usually the only parameter needed for our applications. The sum of the displacements of the two microspheres when a force is applied is Δ*x* = Δ*x*_1_ + Δ*x*_2_ *= γ*_*1*_ *F + γ*_*2*_ *F = γF*. We will discuss a method for checking the assumed linearity of this relationship.

When a single DNA molecule is stretched between the two trapped microspheres, as illustrated in Figure 1C, the separation between the centers of the two traps is

$$d=x+\left(\Delta x_{1}+\Delta x_{2}\right)+\left(r_{1}+r_{2}\right)$$ (3)

where *x* is the end-to-end extension of the DNA, Δ*x*_1_ and Δ*x*_2_ are the displacements of the microspheres from the trap centers, and *r*_1_ and *r*_2_ are the radii of the microspheres. When *d* = *β*(*V*_*mirror*_−*V*_*overlap*_) and *x*/*L*= *A* + *BF* are substituted into Eq. (3) we obtain

$$\beta \left(V_{mirror}-V_{overlap}\right)=\left(LB+\text{γ}\right)\text{ F}+\left(LA+r_{1}+r_{2}\right)\;$$ (4)

This equation has a linear form in which *βV*_*mirror*_ is experimentally controlled and *F* is measured. Thus, linear fits can be used to determine the slope *η* = *γ + LB* and thereby the series compliance *γ = η-LB*, since *L* and *B* are known.

### Experimental Results for Trap Compliance

**FIGURE 2:**
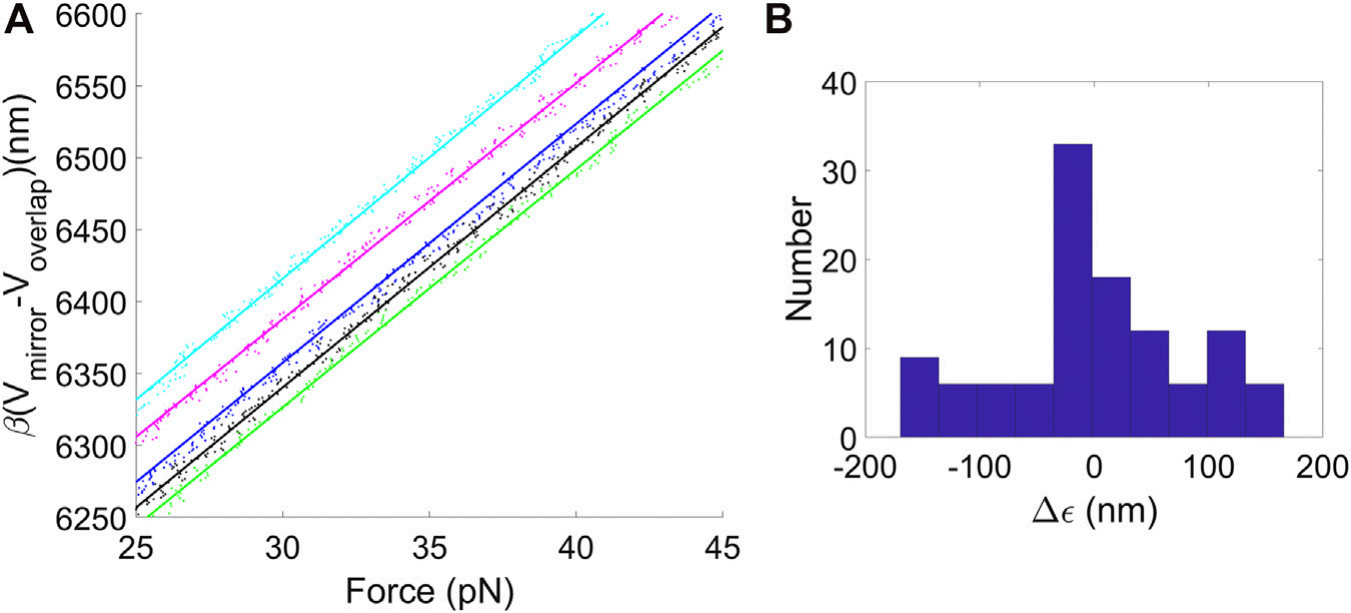
**(A)** Examples of plots of separation between the traps *d* = *β*(*V*_*mirror*_−*V*_*overlap*_) vs. F for data recorded when stretching DNA molecules between the two optically trapped microspheres. The points are experimental measurements and the lines are fits to Eq. (4), used to determine trap compliances, microsphere size variations, and average displacement offset factor. **(B)** Histogram of variations in *ϵ* values determined by the linear fits of the DNA stretching data to Eq. (4), which characterizes the effect of variations in microsphere sizes.

We prepared 10.7 kilobasepair (kbp) DNA molecules with one end biotin labeled for attachment to streptavidin-coated polystyrene microspheres (∼2.1 *μ*m diameter; Spherotech) and the other end labeled with digoxygenin for attachment to anti-digoxygenin coated microspheres (∼2.3 *μ*m diameter; Spherotech). These are commonly used, non-covalent attachments and details are given in the Supplementary Materials and prior publications (Fuller et al., 2006; Rickgauer et al., 2006; delToro and Smith, 2014; delToro, 2015). We recorded N = 99 stretching measurements in which *V*_*mirror*_ was varied and *F* was measured. Examples shown in Figure 2A confirm the dependence is linear as expected. Each dataset was fit to Eq. 4 to determine series compliance *γ* = *η*–LB, yielding an average value *γ* = 12.8 nm/pN (standard deviation = 0.56 nm/pN). Additional measurements were done with a 25.3 kbp DNA and yielded a consistent value *γ* = 12.5 nm/pN (standard deviation = 1.8, N = 180).

Sources of error in determining *γ* include (details are given in Supplementary Materials): 1) 0.2% uncertainty due to uncertainty in the value of *P* reported by Wenner et al., (2002); 2) 3.8% due to uncertainty in S (Wenner et al., 2002); 3) 1.5% due to uncertainty in force calibration factor (*α*) (delToro and Smith, 2014; Wenner et al., 2002); 4) 0.95% due to uncertainty in trap displacement calibration factor (*β*); 5) 0.96% error due to the linearized DNA elasticity approximation (Eq. 2); 6) 0.3% due to noise in the force measurement. Here (1–5) are systematic errors. They are not independent, but together contribute a maximum uncertainty of ∼6%. Although (6) considers a source of random (measurement) error, the actual measured standard deviation is higher (e.g., 4.3% for the 10.7 kbp DNA). While this is acceptably small uncertainty for our applications it suggests there are additional random error sources. Our intuition is these include factors such as the small variations in microsphere size, possibly small variations in microsphere shape, and variations in DNA elasticity due to degradation (such as occasional nicks, i.e. single-stranded breaks) which can occur in individual molecules.

### Microsphere Size Variations

The *y*-intercept of Eq. (4) is *ϵ* = *LA* + *r*_1_ + *r*_2_. *L* and *A* are constants, but the microsphere radii *r*_1_ and *r*_2_ vary by small amounts. This is undesirable since it causes errors in DNA length measurements. The *ϵ* values determined by the linear fits can be used to characterize these variations. A histogram of *ϵ* values, with the mean subtracted, is shown in Figure 2B. The standard deviation is 144 nm. A similar standard deviation of 138 nm was determined from the measurements with the 25.3 kbp DNA. These values are consistent with the standard deviations in the microsphere radii of ∼140 nm reported by the manufacturer. Below we will discuss methods for correcting for these errors.

A related question is whether the differences in microsphere size cause detectible differences in trap compliances. In regime we operate, where microsphere diameter is larger than the trapping laser focal spot, a larger microsphere could cause larger trap compliance (Bormuth et al., 2008). To investigate if this effect is significant, we checked to see if there was any correlation between *ϵ* and γ values in the ensemble of individual measurements. We did not find a significant correlation (correlation coefficient = −0.2). This is likely because the standard deviation in the microsphere size is only ∼7%, so any effect is too small to measure.

### Average Displacement Offset Factor

A simplified method can be used when neglecting the small variations in microsphere sizes is acceptable. Instead of considering *V*_*overlap*_, we define *V*_*contact*_ to be the value of the mirror control voltage *V*_*mirror*_ when the two microspheres come into contact. In principle this could be detected by measuring the force signal when the two microspheres touch, but we find this can be inaccurate due to optical cross-talk/interference between the beams and transverse offsets in trap positions (delToro and Smith, 2014). Instead, one can determine this parameter from the DNA measurements. The value *βV*_*contact*_ = *βV*_*overlap*_ + (*r*_1_ + *r*_2_), so Eq. (4) an be rewritten as

$$\beta V_{mirror}=\left(LB+\text{γ}\right)\text{F}+\left(LA+\beta V_{contact}\right)$$ (5)

Measurements of *ϵ = LA + βV*_*contact*_ allow one to determine values of *V*_*contact*_, since *L*, *A*, and *β* are known. Individual measurements depend on the individual microsphere sizes but from an ensemble of measurements an average value ${\bar{V}}_{contact}$ is determined. The imposed DNA extension is then given by

$$x=\beta \left(V_{mirror}-{\bar{V}}_{contact}\right)-\text{ γF}$$ (6)

which can be controlled since all the variables are known or measured.

### Correction for Microsphere Size Variations

Variation in the trap separation determination caused by variations in microsphere sizes causes error in DNA length measurements. Above we determined a standard deviation of 144 nm in *r*_1_ + *r*_2_. Here we describe methods to correct for this error. While in many types of biophysical studies only relative changes in DNA/biopolymer length may be of interest, in some cases it is desirable to determine absolute length (Keller et al., 2018; Mo et al., 2020).

As an example, we discuss studies of motor-driven viral DNA packaging. We attach a viral procapsid-motor complex to one microsphere and a DNA molecule is translocated into the procapsid by the motor (Keller et al., 2018; Ortiz et al., 2018). We attach the other end of the DNA to a second trapped microsphere, such that the motor pulls the two microspheres together as the tethered DNA is translocated. The length of DNA packaged into the procapsid is equal to the full DNA substrate minus the unpackaged DNA between the two microspheres. Since the operation of the motor and rate of DNA translocation is affected by the length of DNA packaged in the procapsid (Berndsen et al., 2015) we want to measure the absolute DNA length.

One of the viruses we study, bacteriophage phi29, has a 19.3 kbp genome (∼6600 nm), so uncertainty of 144 nm would cause uncertainty of 2.2% in the determination of the fraction of the genome packaged. However, since the motor has a relatively slow translocation rate (maximum of ∼180 bp/s in the conditions we use), a simple method to reduce this error is to use the measured starting tether length as a reference. Packaging is initiated by moving the microsphere carrying DNA near a second trapped microsphere carrying procapsid-motor complexes. The time delay between initiation of packaging and start of data recording is ∼0.2–0.8 s. With a packaging rate of ∼180 bp/s, ∼36–144 bp is packaged during this time, corresponding to ∼12–50 nm, yielding an uncertainty of ∼38 nm. Since this is less than that of ∼144 nm caused by microsphere size variations, use of the measured starting DNA length as a reference in each measurement should be useful for improving accuracy.

To demonstrate this, we analyzed a dataset of N = 60 phage phi29 packaging events where the DNA translocation rate was ∼180 bp/s and found a standard deviation in measured starting lengths of ∼175 nm. This is roughly consistent with the expected uncertainty of ∼144 nm due to microsphere size variations plus ∼38 nm due to variations in initial length of DNA packaged, which implies that subtracting the initial starting length reduces the uncertainty in measurement of absolute length of DNA from ∼175 nm to ∼38 nm. A limitation of this technique is that it would not be beneficial if the DNA translocation rate was so fast that the error caused by the uncertainty in the time delay between initiation and data recording was larger than the error caused by the variation in microsphere sizes.

A second method is based on defining a minimum separation where the two trapped microspheres nearly touch as a reference. This method can be used in cases where DNA translocation proceeds for long enough to bring the microspheres into near contact, or if they can be moved together after the measurement. To test this, we conducted measurements in which we brought two microspheres together into near contact. They are observed using a video imaging system described in the next section. Each microsphere appears as a bright spot surrounded by a dark circular ring. We defined the minimum separation reference as the point where the two dark rings were first observed to touch as the separation was decreased. We then recorded the value of the control voltage, $V_{mirror}^{'}$, and repeated this measurement with N = 32 different pairs of microspheres. The standard deviation in the inferred relative positions was 136 nm, which is close to the estimated uncertainty of 144 nm in *r*_1_ + *r*_2_ determined from the DNA stretching measurements discussed above. This indicates that if $\beta V_{mirror}^{'}$ is recorded for each pair of microspheres these can be used to correct length measurements to reduce error caused by microsphere size variations.

### Checking System Response Linearities

Equation 4 assumes several instrument response relationships are linear. We expect these to be valid based on the system design, but describe here how they can be checked by analysis of the DNA stretching data.

**FIGURE 3:**
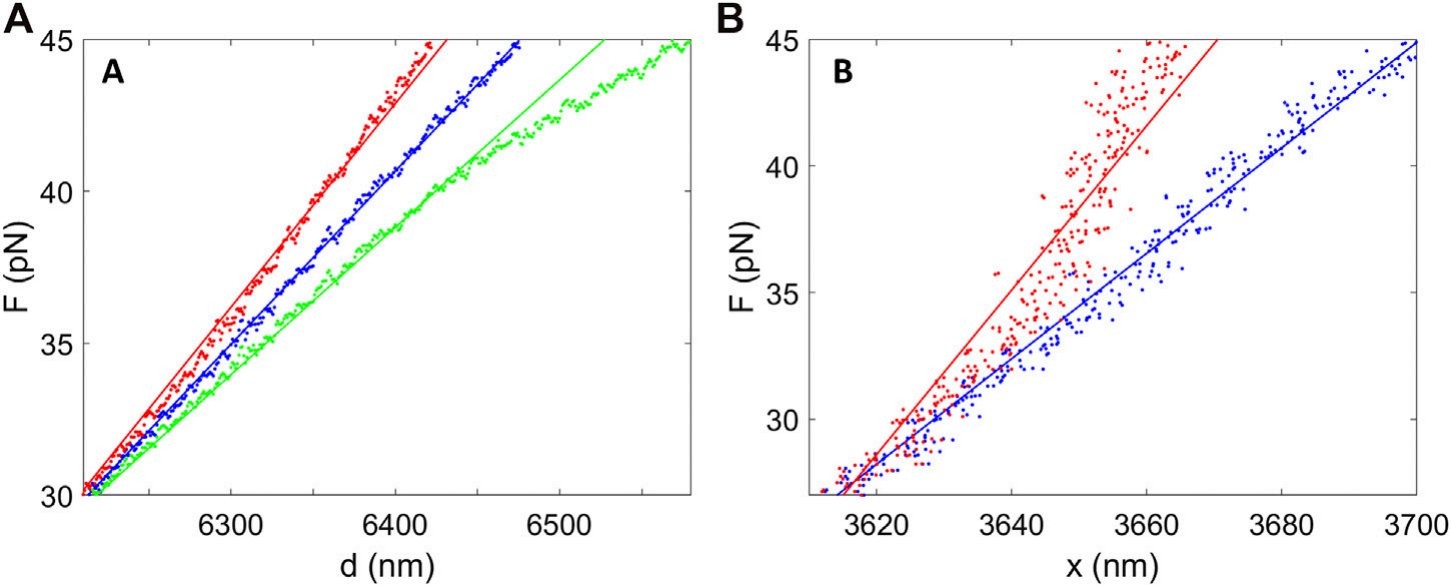
Analyses of the predicted effects of nonlinear errors in system responses. **(A)** The blue points are recorded force vs. separation data and the blue line shows a linear fit, which describes the data well. The red points predict the effect of a quadratic error term affecting the separation control, which causes detectible curvature in the plot. The red line is a linear fit to these points. Similarly, the green points predict the effect of a quadratic error term affecting the force determination, which again causes detectible curvature. The green line is a linear fit to these points. **(B)** The blue points are recorded force vs. DNA extension measurements and the blue line shows a linear fit. The red points predict the effect of a quadratic error in the displacement of the microspheres from the trap centers (Δ*x*), which causes detectible curvature, and the red line is a linear fit to these points.

Trap separation is assumed to obey *d* = *β*(*V*_*mirror*_−*V*_*overlap*_), where *V*_*mirror*_ is the control voltage. This is expected since the trapping beam is steered by a feedback-controlled piezo-actuated mirror. However, the DNA stretching measurements provide a check. Suppose there was a nonlinear response in which the actual relationship deviated from the assumed one by a quadratic term, so that *d*_*actual*_ = *d*_*assumed*_ + *δ*_1_(Δ*d*)^2^, where *δ*_1_ is a constant and Δ*d* is the separation change. The actual DNA extension would be greater than assumed based on the linear relationship, but this would cause the force at each value of the assumed extension to be higher than predicted by the DNA force-extension relationship. The *F* vs. *d* plot is predicted to be linear over 25–45 pN but the error term would cause curvature. That our data does not show significant curvature (Figure 2A) suggests there is no significant error of this type. To quantify the effect of such an error, we subtract the error term *δ*_1_(Δ*d*)^2^ from the plotted extension values and keep the measured force values unchanged. As an example, assume the error increases from zero at F = 25 pN to 15% of Δ*d* at F = 45 pN. This results in simulated data plot where curvature is resolvable within the experimental noise (Figure 3A). That curvature of this magnitude is not observed in the actual data implies that an error of this magnitude does not occur. A limitation is that this method only probes a narrow range of trap separations. Below we discuss a method using microsphere imaging to test a much wider range of trap separations.

Force measurement is assumed to obey *F = αV*_*PSD*_, where *V*_*PSD*_ is the detector signal and *α* the force scale factor. This is expected based on the system design, but it is conceivable that a nonlinear error could occur, for example due to optical misalignments. We perform a similar analysis as above to consider an error *F*_*actual*_ = *F*_*assumed*_ + *δ*_1_(Δ*F*)^2^, where *δ*_2_ is a constant and Δ*F* is the change in force. As shown in Figure 3A we again find that if this error increased from zero at 25 pN to 15% of Δ*F* at 45 pN this would cause detectible curvature in the *F* vs. *d* plot, but this is not observed in our recorded data.

Displacement of the microsphere from the trap is assumed to be Hookean, Δ*x = γF*, where *γ* is the compliance, but there could be deviations. Some studies find that beyond a low-force Hookean regime compliance decreases slightly, before ultimately increasing again at the highest forces when the bead begins to escape the trap (Richardson et al., 2008; Jahnel et al., 2011). If this occurred and was neglected, it would cause the measured forces in the force vs. DNA extension (*x* = *d* − Δ*x*) plot to be higher than predicted. We find that a quadratic error term of 15% maximum magnitude in Δ*x* would cause detectible curvature, but curvature of this magnitude is not observed (Figure 3B).

### Checking Trap Positioning Linearity

The DNA force-extension measurements described above provided a test of the validity of the trap separation control linearity *d* = *β*(*V*_*mirror*_−*V*_*overlap*_), but only over a limited range of separations. To check that the relationship is valid over the full range one can also use video imaging and tracking of the microsphere centroid.

**FIGURE 4:**
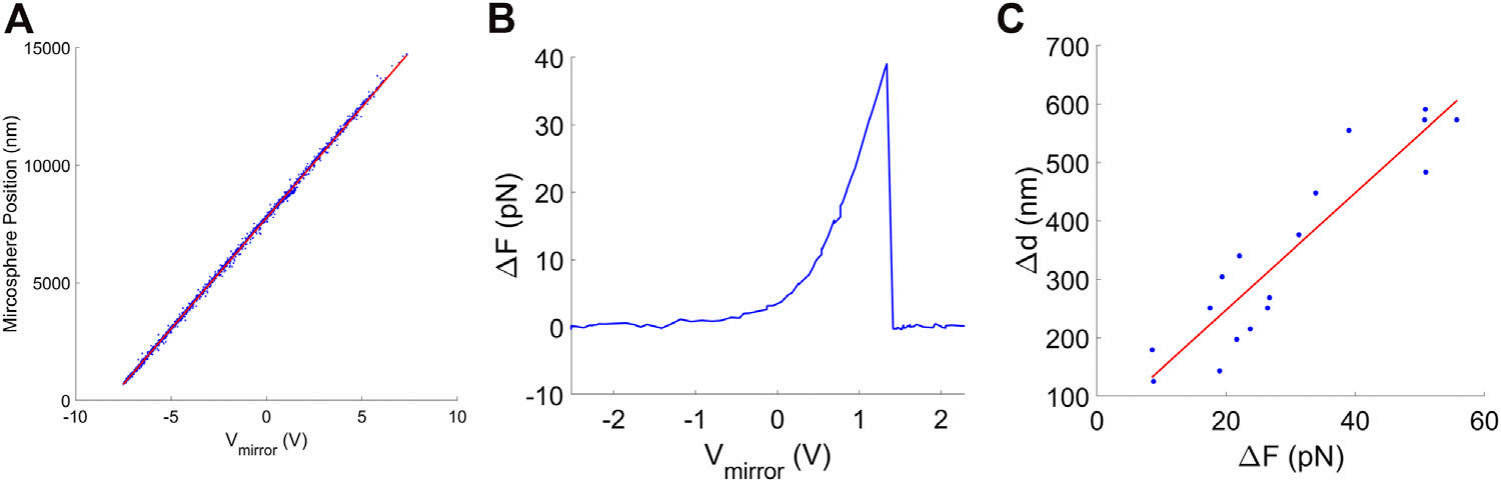
**(A)** Microsphere position, determined by image centroid tracking, vs. mirror control signal (blue points). The red line shows a linear fit. **(B)** Example of a measured DNA detachment event where the separation of the traps is increased (by increasing *V*_*mirror*_) and the force is measured to suddenly drop to zero. **(C)** Measurements of the movement of the microsphere back to the trap center, determined by image centroid tracking, after DNA detachment events that occurred at different force levels. The red line is a linear fit to the data.

We recorded the image formed by the upstream microscope objective with a video camera and a video capture card (NI-PCI-1405, National Instruments, Inc.) (see Supplemental Material). The centroid of the microsphere was tracked by locating the dark circular ring in the microsphere image with the “imfindcircles” function in the Matlab Image Processing Toolbox, which employs a circular Hough transform algorithm. Pixels in the video image were converted to nm using the known value of *β*. In this manner we could confirm that trap position is linearly proportional to mirror-tilt control voltage over the full range of ∼13 μm (Figure 4A).

### Individual Trap Compliances

Fitting DNA stretching data to Eq. (4) described above provides a convenient way to determine the series compliance of the traps, which is sufficient for most of our studies since one can determine the change in DNA extension. However, some studies may require knowledge of individual trap compliances (Moffitt et al., 2006). This can be done by combining microsphere imaging with DNA stretching to apply controlled forces. It can be challenging to determine the compliance of a movable trap because a small displacement due to force needs to be discerned from a much larger imposed movement of the trap. A method described below provides a solution to this issue.

When high forces are applied to stretch the DNA after some time the molecule detaches due to force-induced dissociation of the digoxygenin-anti-digoxygenin linkage (Fuller et al., 2006). An example is shown in Figure 4B. The DNA was stretched by increasing the trap separation in small steps but it suddenly detached at *F* ∼ 40 pN, causing the force to drop to zero. Such events can be used to determine the trap compliance because the separation between the traps remains constant while the microsphere suddenly moves a distance Δ*x*_1_ = *d γ*_*1*_Δ*F*, where *γ*_*1*_ is the trap compliance and Δ*F* is the force drop. Because detachments occur randomly at different forces ranging from ∼5 to 50 pN, the predicted relationship Δ*x*_1_ = *γ*_*1*_Δ*F* can be tested over a wide force range to confirm a Hookean response and determine *γ*_*1*_. Examples of such measurements are shown in Figure 4C and are consistent with an assumed linear Δ*x*_1_ = *γ*_*1*_Δ*F* relationship. The value of *γ*_*1*_ we obtain by a linear fit to these data is 10.9 ± 1.1 nm/pN. The significant scatter in the data points is attributable to the low resolution of our imaging system, which was originally only designed to allow the user to check for the presence of a microsphere. Since methods have been developed to measure microsphere movements with nanometer-level resolution (Ueberschär et al., 2012), the method has potential to be improved significantly.

## Conclusion

We demonstrated methods by which trap compliance can be determined in a robust matter via measurements of DNA stretching in the regime where a linear elasticity approximation is valid. This method is especially useful if one is already working with DNA, but more generally provides an independent method to confirm other calibration methods in the literature (Smith et al., 2003; Berg-Sørensen and Flyvbjerg, 2004; Neuman and Block, 2004; Tolić-Nørrelykke et al., 2006; Viana et al., 2006; Jahnel et al., 2011; Sarshar et al., 2014; Jones et al., 2015; Bui et al., 2018; Yale et al., 2018; Melo et al., 2020). In comparison to many other methods, it does not require a calibrated imaging/optical system, fluid flow, or precise sample stage control, and four calibration parameters (force scale, trap displacement scale, relative trap position, and compliance) can be simultaneously determined. Calibration extends to high forces (45 pN) and the linearity of system responses can be tested. The method is also useful for characterizing and correcting for the effect of variations in microsphere sizes on extension measurements. Finally, we show that combined microsphere imaging can be further used to check individual trap positions and compliances.

## Data Availability

The raw data supporting the conclusions of this article will be made available by the authors, without undue reservation.
