# Supplementary material for: Determining Trap Compliances, Microsphere Size Variations, and Response Linearities in Single DNA Molecule Elasticity Measurements with Optical Tweezers
Source: Front Mol Biosci. 2021 Mar 22;8:605102. doi: 10.3389/fmolb.2021.605102 (PMC8019724; doi:10.3389/fmolb.2021.605102)
Supplement: Supplementary file 1 [file datasheet1.pdf]

Supplementary Materials for "Determining Trap Compliances, Microsphere Size Variations, and Response Linearities in Single DNA Molecule Elasticity Measurements with Optical Tweezers", by Y. Mo et al.

Optical Tweezers Instrument

A schematic diagram of the instrument is shown below and additional details on the design and construction are given in Ref 21. A 1064-nm linearly polarized CW fiber laser (IPG Photonics, Inc.) is expanded and collimated by a telescope (T_1) before being split into two orthogonal polarizations by a polarizing beam splitter (PB_1). One beam is reflected by a stationary mirror, while the other reflects off a mirror tilted by a computer-controlled piezoelectric actuator (PM). The two beams are recombined to follow the same path by a second polarizing beam splitter (PB_2) and relayed by a second telescope (T_2), to place the plane of the rotating mirror conjugate to the back focal plane of a 60x 1.2-NA water-immersion objective (O_1; Olympus, Inc.), so that steering the mirror moves one trap in the sample plane. This objective focuses the beams to form the two traps in the fluid chamber (FC), each ~150 mW in the sample plane. An identical objective (O_2) collects the exiting beams and a final polarizing beam splitter (PB_3) separates the beams by polarization so that each is directed to a position-sensing detector (PSD_1 and PSD_2, On-Trak, Inc). Relay lenses place the back focal plane of O_2 conjugate to the detector surface so that the PSDs signals are proportional to the beam deflections and thus the transverse forces acting on the microspheres. A ~10 mW blue LED ("light source"), imaged onto the back focal plane of O_2 illuminates the sample and objective O_1 images the sample plane onto a CCD video camera (Watec WAT-902H2).

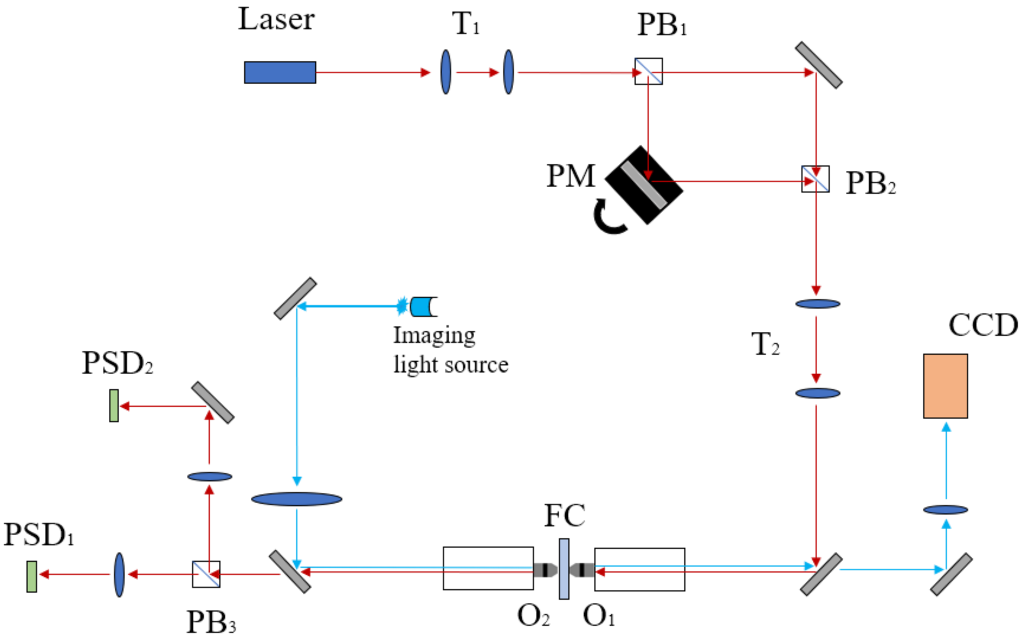

Determining force and displacement scale factors

The force scale factor α is determined by measuring the DNA overstretch transition. As described in detail in Ref. 22, we measure the PSD voltage at which this occurs by finding the midpoint (which corresponds to F=64 pN) between the intersection points of a linear fit to the overstretch plateau and two polynomial fits to the sections of data before and after the overstretch transition plateau. The displacement scale factor β is determined as described in detail in Ref. 22. In brief, the values of V_{mirror} that correspond

to the value $F = 33.4$ pN, where $x = L$, were determined for the two different DNA construct lengths (V_1 and V_2). At this force Eq. (6) gives $\beta = (L_2 - L_1)/(V_2 - V_1)$, where L_1 and L_2 are the known contour lengths of the two DNA constructs.

Preparation of DNA constructs and microspheres

DNA molecules were prepared by PCR from lambda phage DNA (NEB, Inc.) using biotin and anti-digoxygenin labeled primers (IDT DNA, Inc.) using the methods in Ref. 41. The 10.7 kbp DNA construct used the forward primer Biotin-5'-CATCATCATGCAGAACATGCGTGACGAAGAGCTG-3' and the reverse primer dig-5´-ATACGCTGTATTCAGCAACACCGTCAGGAACACG-3'. The 25.3 kbp DNA construct used the forward primer Biotin-5'-CTGATGAGTTCGTGTCCGTACAACTGGCGTAATC-3' and the reverse primer dig-5'-ATCCGATCTGCGTTACCGAATGGATGGATG-3'. DNA-coated microspheres were prepared by incubating 10 μL of 0.5% w/v 2.1 μm streptavidin-coated microspheres (Spherotech, Inc.) in 1x PBS (Phosphate Buffered Saline) with ~70 ng of DNA and 20 μg BSA (Bovine Serum Albumin) (Sigma-Aldrich, Inc.) for 20 minutes. Digoxygenin-coated microspheres were prepared by incubating 5 μL of 5% w/v 2.3um protein G-coated microspheres (Spherotech, Inc.) suspended in 1x PBS with 200 ng of anti-digoxygenin (Sigma-Aldrich, Inc.) for 45 minutes, as described in Ref. 41 (also see: PhD Thesis of Nicholas Keller (Univ. of California, San Diego, 2016)).

DNA Force-Extension Measurements

Multiple force-extension curves for the two different DNA construct lengths were collected. Tethers were formed by briefly bringing the trapped DNA- and anti-dig-coated microspheres together and then separating them while checking for the increase in PSD signal indicative of increasing force on the microspheres. Once a DNA tether was formed, a force-extension curve was measured by increasing V_{mirror} at a fixed rate of 500 nm/s between values V_1 and V_2, where V_1 is the largest control voltage such that the PSD signal does not differ from background, and V_2 corresponds to a microsphere separation distance large enough that the DNA tether will either detach or complete an overstretch transition. PSD data were recorded at a rate of 1 kHz. The background PSD signal as a function of V_{mirror} was also subtracted from each force-extension dataset.

Error in the determination of the series trap compliance

We analyzed uncertainties/error sources in the determination of the compliance parameter γ by considering the effect of various factors on the use of Equation (4) to fit the DNA force-extension datasets:

i) Wenner et al. (Ref. 35) report an uncertainty of 2 nm in the DNA persistence length (P), which through our Equations (1) and (2) results in an uncertainty of 0.9% in the parameter B. Through fits of Equation (4) this results in an uncertainty of 0.2% in the determination of γ.

ii) Wenner et al. (Ref. 35) report an uncertainty of 217 pN in the DNA stretch modulus (S), which results in an uncertainty of 11.6% in the parameter B. Through Equation (4) this results in an uncertainty of 3.8% in the determination of γ.

iii) Uncertainty in the force measurements is caused by an uncertainty of 2% in the force calibration factor α, due to uncertainty in the DNA overstretch transition force plateau reported by Wenner et al. (Ref. 35) and random measurement errors in our overstretch transition measurements (as discussed by delToro et al. (Ref. 22)). Through Eq. (4) this uncertainty results in an uncertainty of 1.5% in the determination of γ.

iv) Uncertainty in the trap displacements is caused by an uncertainty of ~0.7% in calibration factor β as discussed by delToro et al. (Ref. 22). Through Eq. (4) this uncertainty results in an uncertainty of 0.95% in the determination of γ.

v) Analysis of Equations (1), (2), and (4) indicate that the approximation that the DNA force-extension relationship is linear in the range from 25-45 pN, when it actually has a small nonlinearity, causes an error of 0.96% in the determination of γ.

Note: The five sources of uncertainty listed above do not have completely independent effects. By systematically varying all these parameters together, we calculated that the maximum induced uncertainty in gamma is ~6%.

vi) Finally, we considered the effect of noise in the force measurements, due to Brownian and instrumental noise. We investigated this by generating an ensemble of simulated datasets with the DNA force-extension law fixed according to Eq. (1) but with random Gaussian noise added to each dataset. The force noise level in these simulated datasets was set equal to that in the measured datasets. By fitting these simulated datasets to Eq. (4) we determined that this noise causes 0.3% uncertainty in the determination of γ.
